# Supplementary material for: Birds of a feather moult together: Differences in moulting distribution of four species of storm-petrels
Source: PLoS One. 2021 Jan 22;16(1):e0245756. doi: 10.1371/journal.pone.0245756 (PMC7822297; doi:10.1371/journal.pone.0245756)
Supplement: S1 File — (DOCX) [file pone.0245756.s001.docx]

**Birds of a feather moult together: differences in moulting distribution of four species of storm-petrels**

Anne N.M.A. Ausems^1^, Grzegorz Skrzypek^2^, Katarzyna Wojczulanis-Jakubas^1^, Dariusz Jakubas^1^

^1^The University of Gdańsk, Faculty of Biology, Department of Vertebrate Ecology and Zoology, ul. Wita Stwosza 59, 80-308 Gdańsk, Poland

^2^The University of Western Australia, West Australian Biogeochemistry Centre, 35 Stirling Highway, Crawley WA 6009, Australia

Corresponding author: Anne N.M.A. Ausems, anne.ausems@gmail.com

**Supporting Information 1: Base maps for stable oxygen and carbon ocean isoscapes and chlorophyll-*a* concentrations**

For the *isocat* analyses (Campbell, 2020) we used prediction isoscapes for both *δ*^18^O and *δ*^13^C (Fig. S1). We used seasonally averaged plankton *δ*^13^C prediction isoscapes provided by C. Trueman from models described in Magozzi et al. (2017), for the core non-breeding periods of the northern (November to March) and southern (May to October) species separately. For *δ*^18^O we used an annually averaged gridded dataset for Global Seawater Oxygen-18 Database isoscape obtained from LeGrande and Schmidt (2006; <https://data.giss.nasa.gov/o18data/>) and visualized in ArcMap 10.3.1 (ESRI, 2014). For the two northern species, we only used data from the Atlantic Ocean as the studied populations do not migrate to other oceans and thus, we restricted the rasters to the area between 75 °W and 52 °E.

To validate the moulting areas predicted by the *isocat* analyses, we used chlorophyll-*a* concentrations as a proxy for food abundance. We used chlorophyll-*a* concentrations at the surface layer from remote sensing MODIS Aqua satellite data (NASA Ocean Color Web, <https://oceancolor.gsfc.nasa.gov/>). We created concentration rasters for the corresponding core non-breeding periods for the species from the northern (November to March 2003-2018) and southern (May to October 2003-2018) hemispheres (Fig. S1). We averaged monthly maps in ArcMap 10.3.1 (ESRI, 2014).

**References**

Campbell, C., 2020. isocat: Isotope Origin Clustering and Assignment Tools.

ESRI, 2014. ArcMap 10.3.1.

LeGrande, A.N., Schmidt, G.A., 2006. Global gridded data set of the oxygen isotopic composition in seawater 33, 1–5. https://doi.org/10.1029/2006GL026011

Magozzi, S., Yool, A., Vander Zanden, H.B., Wunder, M.B., Trueman, C.N., 2017. Using ocean models to predict spatial and temporal variation in marine carbon isotopes. Ecosphere 8. https://doi.org/10.1002/ecs2.1763


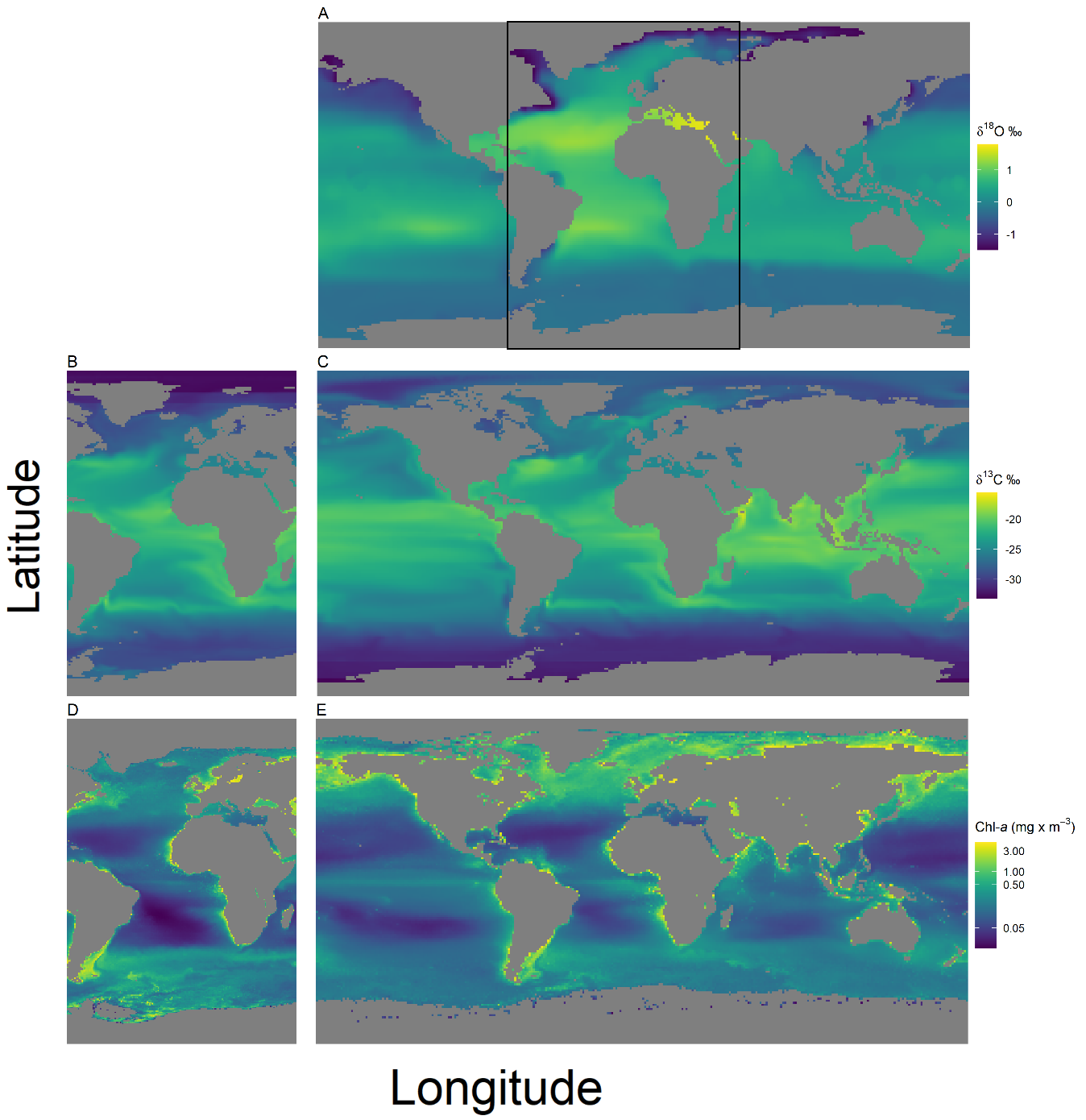


Figure S1 Base maps for stable oxygen and carbon ocean isoscapes and chlorophyll-a concentrations: Panel A – Annually averaged δ^18^O isoscape; panel B – Seasonally averaged plankton δ^13^C isoscape for the Northern species (non-breeding; November – March); panel C – Seasonally averaged plankton δ^13^C isoscape for the Southern species (non-breeding; May – October); panel D – Chlorophyll-a concentrations for the non-breeding period of the Northern species (non-breeding; November – March); panel E – Chlorophyll-a concentrations for the non-breeding period of the Southern species (non-breeding; May – October). The black rectangle panel A shows the area between 75 °W and 52 °E used for the Northern species. Panel B and D are restricted to the same area.
