# Supplementary material for: Birds of a feather moult together: Differences in moulting distribution of four species of storm-petrels
Source: PLoS One. 2021 Jan 22;16(1):e0245756. doi: 10.1371/journal.pone.0245756 (PMC7822297; doi:10.1371/journal.pone.0245756)
Supplement: S2 File — (DOCX) [file pone.0245756.s002.docx]

**Birds of a feather moult together: differences in moulting distribution of four species of storm-petrels**

Anne N.M.A. Ausems^1^, Grzegorz Skrzypek^2^, Katarzyna Wojczulanis-Jakubas^1^, Dariusz Jakubas^1^

^1^The University of Gdańsk, Faculty of Biology, Department of Vertebrate Ecology and Zoology, ul. Wita Stwosza 59, 80-308 Gdańsk, Poland

^2^The University of Western Australia, West Australian Biogeochemistry Centre, 35 Stirling Highway, Crawley WA 6009, Australia

Corresponding author: Anne N.M.A. Ausems, anne.ausems@gmail.com

**Supporting Information 2: European storm-petrel results including two outliers**

The CIT terminal nodes did not differ significantly in *δ*^13^C (Welch two-sample *t*-test; *t*_62.8_ = 1.68, p = 0.097) but CIT node 2 had significantly higher *δ*^18^O values than CIT node 3 (*t*_52.6_ = 5.57, p < 0.001; Fig. S2.1; Table S2.1).

The similarity in the scaled probability-of-origin distribution maps was very low (Jaccard index; J = 0.050; Fig. S2.2).

Chlorophyll-*a* concentrations in CIT node 2 were significantly lower in the areas with the 76 % – 100 % highest scaled probability-of-origin values than in the lower 0 % – 75 % value areas (Welch two sample *t*-test; *t*_215.9_ = 9.32, p < 0.001), but did not differ between scaled probability-of-origin areas in CIT node 3 (*t*_254.5_ = 1.13, p = 0.260).


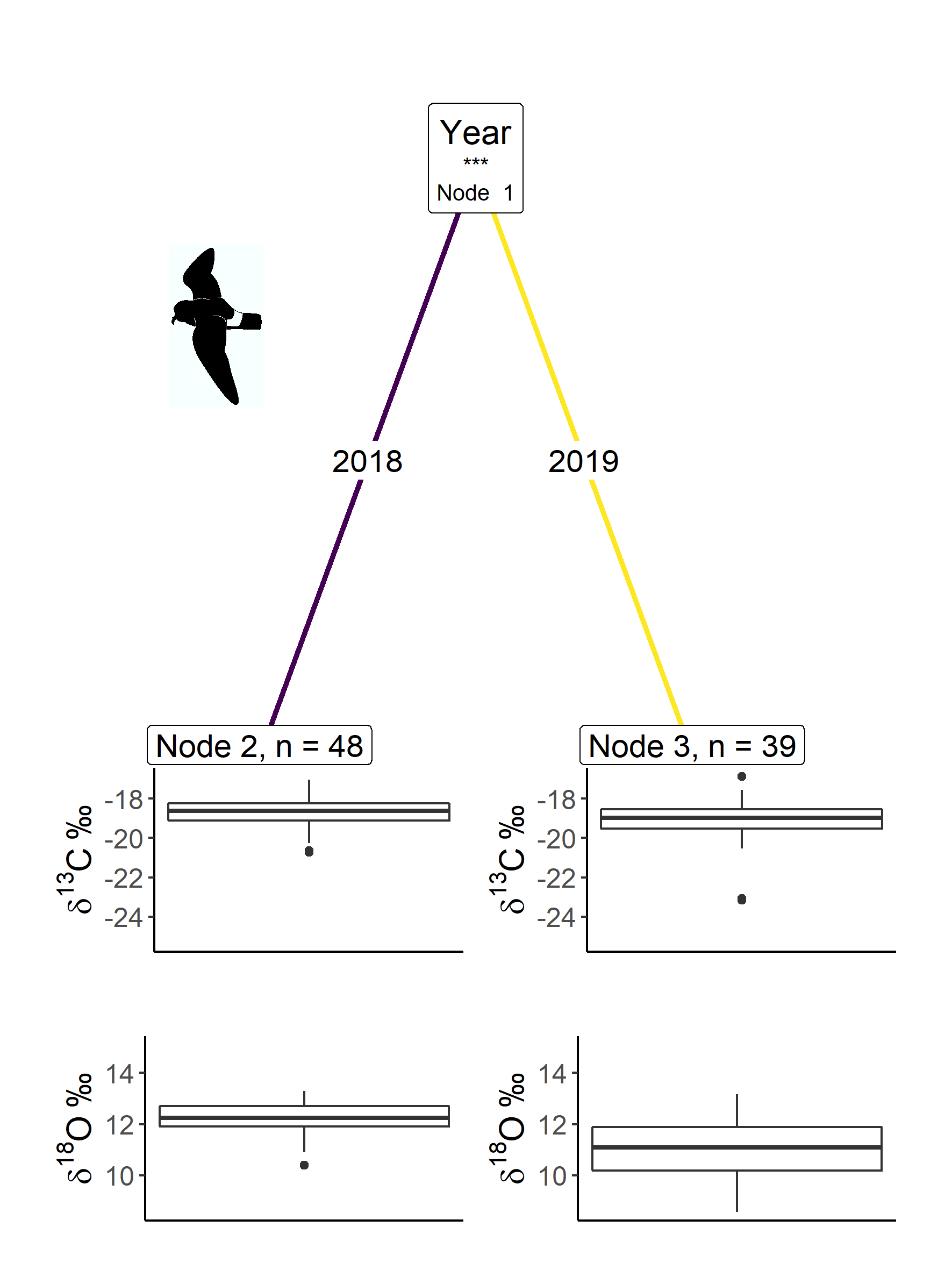


Figure S2.1 Conditional inference trees (CIT) characterizing factors affecting the stable carbon (δ^13^C) and oxygen (δ^18^O) isotopic signatures for the European storm-petrel including outliers during the moulting period. We used Species, Year, δ^15^N (stable nitrogen isotope composition), FGR (feather growth rate), BM (body mass), TL (tarsus length), and WL (wing length) as initial predictors. Body morphometrics (i.e. BM, TL and WL) were measured during the breeding season after moulting. Only variables with a significant dividing effect are shown in order of importance from the top down. At each node the dividing variable and corresponding p-value sign are listed in the box. These significance levels represent the test of independence between the listed variable and the response variables. Terminal CIT nodes indicate variable levels characterizing the response variable. Density plots above node boxes show the distribution of the continuous divisive variables, with the cut-off point dividing the colours. Boxplots show the median (band inside the box), the first (25%) and third (75%) quartile (box), the lowest and the highest values within 1.5 interquartile range (whiskers) and outliers (circles). ESP – European storm-petrel; n – number of individuals in each terminal CIT node group. P-values < 0.001 are shown with ***, p-values < 0.01 are shown with ** and p-values < 0.05 are shown with *.

Table S2.1 The mean ± SD *δ*^13^C and *δ*^18^O values of the subgroups distinguished based on the conditional inference tree (CIT) terminal nodes for the European storm-petrel including outliers. The individuals were divided into groups with differing *δ*^13^C and *δ*^18^O values, based on variables described in the text. Terminal node – terminal CIT node number; n – sample size. See also Fig. S2.1 for tree results.

| Terminal node | n | *δ*^13^C_VPDB_  (‰) | *δ*^18^O_VSMOW_  (‰) |
| --- | --- | --- | --- |
| 2 | 48 | -18.8 ± 0.8 | 12.2 ± 0.6 |
| 3 | 39 | -19.1 ± 1.2 | 11.0 ± 1.2 |
| Total | 87 | -18.9 ± 1.0 | 11.7 ± 1.1 |


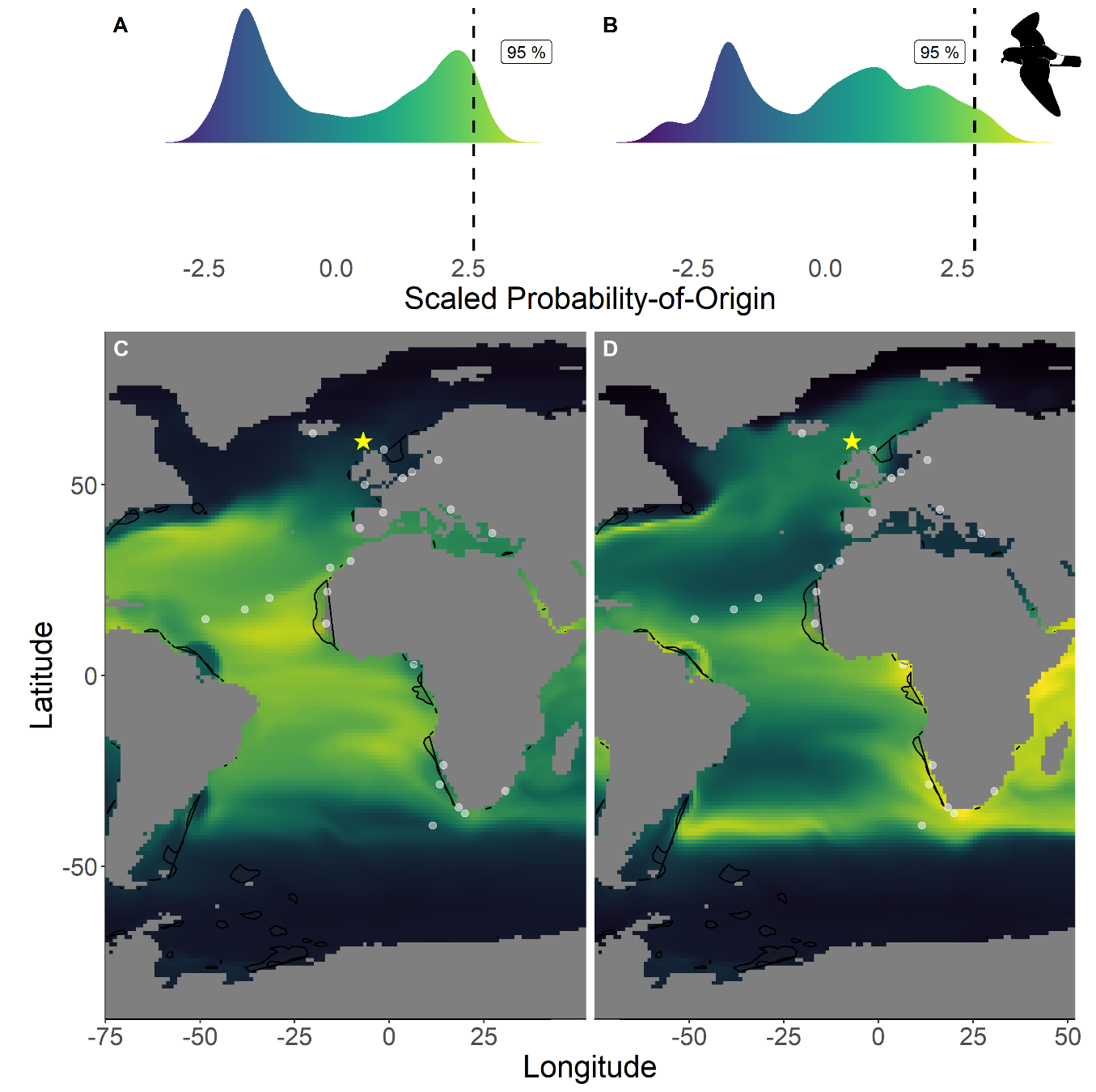


Figure S2.2 Scaled probability-of-origin maps based on δ^13^C and δ^18^O for each group for the European storm-petrel (ESP) including outliers. Terminal nodes from a conditional inference tree (CIT) based on differences between years, and correlated to body morphology (Fig. S2.1) were treated as groups. Panel A – Scaled probability-of-origin value distribution for terminal CIT node 2; panel B – scaled probability-of-origin value distribution for terminal CIT node 3; panel C – scaled probability-of-origin map for terminal CIT node 2; panel D – scaled probability-of-origin map for terminal CIT node 3. Scaled probability-of-origin values are shown on a relative high (yellow) – low (black) gradient in both the density plots and maps. The 95 % quantile of the scaled probability-of-origin values per terminal CIT node are shown with the dashed line. Shaded contours show high chlorophyll-a concentration areas (upper 95 % of the data), and white dots show observation locations (ebird.com, 2020; Observation.org, 2020). The yellow star indicates the location of the breeding colony where birds were sampled.

Table S2.2 Scaled probability-of-origin and chlorophyll-a concentration values around each observation location per terminal CIT node for the European storm-petrel including outliers. Scaled probability-of-origin and chlorophyll-a concentration values were averaged for a buffer of approximately 10 ° around the average latitude and longitude for each observation location. The 50 % and 95 % quantiles were calculated for the entire raster, for both the scaled probability-of-origin maps and the chlorophyll-a concentration maps. See also Fig. S2.2 for scaled probability-of-origin distributions. Note: the scaled probability-of-origin values are relative, i.e. not comparable between species from both hemispheres.

|  | Scaled probability-of-origin | | | Chlorophyll-*a* | | |
| --- | --- | --- | --- | --- | --- | --- |
| Terminal node | Mean ± SD | 50 % | 95 % | Mean ± SD | 50 % | 95 % |
| 2 | 1.85 ± 0.38 | -0.17 | 2.60 | 1.33 ± 4.23 | 0.24 | 1.34 |
| 3 | 1.99 ± 0.64 | 0.33 | 2.82 |  |  |  |

Table S2.3 Mean ± SD of the scaled probability-of-origin values per marine eco-realm per terminal CIT node for the European storm-petrel including outliers. Marine eco-realms were defined in Spalding et al. (2007). Mean scaled probability-of-origin values were compared to the respective 50 % and 95 % scaled probability-of-origin quantiles per terminal CIT node per species (Table S2.2). Mean scaled probability-of-origin values > 50 % and < 95 % quantiles of the corresponding terminal CIT node are *italicised* and mean scaled probability-of-origin values > 95 % quantile of the corresponding terminal CIT node are **bolded**. See also Fig. S2.2 for scaled probability-of-origin distributions. Note: the scaled probability-of-origin values are relative.

|  | Terminal node | |
| --- | --- | --- |
| Eco-realm | 2 | 3 |
| Arctic | -2.02 ± 0.29 | -2.02 ± 1.06 |
| Central Indo-Pacific | NA ± NA | NA ± NA |
| Eastern Indo-Pacific | NA ± NA | NA ± NA |
| Southern Ocean | -1.61 ± 0.24 | -1.70 ± 0.29 |
| Temperate Australasia | NA ± NA | NA ± NA |
| Temperate Northern Atlantic | *0.30 ± 1.36* | 0.18 ± 0.95 |
| Temperate Northern Pacific | NA ± NA | NA ± NA |
| Temperate South America | *-0.17 ± 0.78* | *0.33 ± 1.24* |
| Temperate Southern Africa | *1.61 ± 0.40* | **2.93 ± 0.37** |
| Tropical Atlantic | *2.17 ± 0.51* | *1.65 ± 0.82* |
| Tropical Eastern Pacific | NA ± NA | NA ± NA |
| Western Indo-Pacific | NA ± NA | NA ± NA |

**References**

ebird.com, 2020. eBird Basic Dataset May 2020.

Observation.org, 2020. Stichting Observation International and local partners. https://www.observation.org

Spalding, M.D., Fox, H.E., Allen, G.R., Davidson, N., Ferdaña, Z.A., Finlayson, M., Halpern, B.S., Jorge, M.A., Lombana, A., Lourie, S.A., Martin, K.D., McManus, E., Molnar, J., Recchia, C.A., Robertson, J., 2007. Marine ecoregions of the world: A bioregionalization of coastal and shelf areas. Bioscience 57, 573–583. https://doi.org/10.1641/B570707
