# Supplementary material for: Birds of a feather moult together: Differences in moulting distribution of four species of storm-petrels
Source: PLoS One. 2021 Jan 22;16(1):e0245756. doi: 10.1371/journal.pone.0245756 (PMC7822297; doi:10.1371/journal.pone.0245756)
Supplement: Attachment — Submitted filename: Response to reviewers 2.docx [file pone.0245756.s005.docx]

Vitor Hugo Rodrigues Paiva	05.01.2021
Academic Editor
PLOS ONE

Dear Dr. Vitor Paiva,

On behalf of all authors, I would like to thank you and the reviewers for the thorough review process and the valuable comments on our manuscript. As after the first round of reviews, we have addressed all comments raised by the reviewers below. We have started each answer with *“Response”*, and included the section, page and line numbers referring to the revised manuscript with changes marked when appropriate.

We hope that our changes are satisfactory and meet the expectations of the PLOS ONE editors and reviewers. Please, do not hesitate to contact me if you have any questions, comments or would like further clarification on anything. Thank you for your time considering our manuscript.

We look forward to hearing from you.

Yours sincerely,

Anne N.M.A. Ausems

**Reviewer #1**

The revision of the manuscript “Birds of a feather moult together: differences in moulting range of four species of storm-petrels” from Ausmes and colleagues addressed most of the reviewers’ comments in a satisfactory manner and as a result, the manuscript has substantially improved (specially the results section). However, I still have a few minor comments.

*Response:* *Thank you for the positive words!*

Abstract
L35. I would specify: “to predict potential moulting areas of the sampled feather type”.

*Response:* *We have changed the sentence accordingly.*

*Abstract; page 2; lines 34 – 35:* […] data to predict potential moulting areas of the sampled feather type.

Introduction
L84-85. This sentence needs to be changed slightly. Maybe something like: “This latter species is considered the world’s most abundant seabird species. However, relatively little is known about storm-petrels ecology during the non-breeding period.”

*Response: We have changed the sentence as suggested.*

*Introduction; page 4; lines 85 – 87:* The latter species is considered the world’s most abundant seabird species. However, relatively little is known about storm-petrel ecology during the non-breeding period.

L100-106. This is a nice and important addition to the manuscript from the previous version.

*Response: We are pleased to hear that.*

Materials and methods
The order in which the information is presented in this section is rather awkward and could use some re-ordering. The authors mentioned number of birds captured and feather collection before the “data collection” section. The field site locations are described outside of the “field study” section.

*Response: To address the concerns raised here, we have changed the section header to “Study species and location”, specified that we captured adults and moved the last paragraph of this section to the next (i.e. under Field Study).*

*Materials and methods; Study species and location; page 6; lines 129 – 132:*
**Study species and location**

We captured ESP and LSP adults in August of 2018 (n = 52; n = 56, respectively) and 2019 (n = 40; n = 37, respectively) on the island of Mykines, Faroe Islands (62°05´N, 07°39´W), and BBSP and WSP adults during the austral summer of 2017 (n = 15; n = 100, respectively) and 2018 (n = 19; n = 126, respectively).

*Materials and methods; Data collection; Field study; page 8; lines 186 – 193:* It took several weeks for the sampled rectrices to be fully grown (ESP 30.6 ± 8.5 d; LSP 40.8 ± 13.0 d; BBSP 18.5 ± 2.5 d; WSP 18.7 ± 3.0 d; [59]). Thus, the rectrix formation period, overlapping to a considerable extent with flight feather moult[14,24], includes a considerable part of the non-breeding period, even if feather growth started at the end of the breeding season. Although sampling tail-feathers increases the chance of sampling a feather moulted during the breeding period in LSP, we considered the uncertainty around the location of the start of tail moult too great to justify adding the negative effect of increasing feather gaps by sampling a more central feather.

L229. Introduce acronyms the first time they are used.

*Response: We have removed this part of the sentence and now refer to ‘the analyses’ in general.*

*Materials and methods; Statistical analyses; page 11; lines 239 - 240:* The results for the analyses including the outliers are reported in the supplementary data (S2).

**Reviewer #2**

I think the manuscript was greatly improved by all the recommendations and tweaks that the authors incorporated from the reviewers. Now the manuscript is clearer, comprehensive from the beginning to the end, and the authors defended very well the rebuttal. I only have some minor things to point:

*Response: Thank you for the positive feedback!*

L26: I cannot make you use the new nomenclature of the LSP, but I still think you should use it – if it is changing, the old one is meant to stop being used. I would suggest using it right from the abstract on, and further down, instead of “been changed to Hydrobates leucorhous” you should write “described before as Oceanodroma leucorhoa”.

*Response: We have followed your advice, and changed the species name in the abstract and introduction to Hydrobates leucorhous, and changed the sentence in the materials and methods to mention the old name.*

*Materials and methods; Study species and location; pages 6 – 7; lines 138 – 140:* The species name of LSP was therefore recently changed by BirdLife from *Oceanodroma leucorhoa* to *Hydrobates leucorhous* [43] though the old nomenclature is still widely used as well.

L191: Add “(PCR)” after “polymerase chain reaction” since it is a very well-known acronym.

*Response: We have included “(PCR)” as suggested.*

L195-196: “(…) we tested the sex of a total of 4 BBSP (2 females; 1 male)”. It sums to 3 birds, not 4.

*Response: You are correct; the absolute total is four but only three were sexed based on the PCR results. We have corrected it.*

L223: Uniform the values (0.1 and 0.5 or 0.10 and 0.50)

*Response: We have added a 0 after 0.5 to make the values uniform.*

L433 and L436: You have empty parenthesis, please complete with what was supposed to come in them.

*Response: These were unfortunately left behind after revising the section. They are removed now.*

L646: “factors ( Fig 5).” Delete the space between parenthesis and Fig.

*Response: We have corrected this accordingly.*

**Reviewer #3**

(No Response)
